# Iatrogenic Compartment Syndrome Secondary to Burn Dressing in a 2-Year-Old Child

**DOI:** 10.1055/s-0039-1698403

**Published:** 2019-10-31

**Authors:** Carlos Delgado-Miguel, Antonio Jesus Muñoz-Serrano, Miriam Miguel-Ferrero, Karla Estefanía Rodríguez, María Velayos, Paloma Triana, Mercedes Diaz, Juan Carlos López-Gutiérrez

**Affiliations:** 1 Department of Pediatric Surgery, Hospital Universitario La Paz, Madrid, Spain

**Keywords:** compartment syndrome, iatrogenic, compressive dressing, burns, children

## Abstract

We report a severe case of compartment syndrome due to a compressive burn dressing. An otherwise healthy 2-year-old girl presented at her local health center with a superficial partial-thickness thermal burn on the dorsum of the mid phalanx of the second finger of her right hand. A compressive dressing was applied solely to the affected finger. Forty-eight hours afterward, the patient presented in the emergency room with severe pain of the finger. After removal of the dressing, a circular constrictive eschar was observed at the base of the finger, secondary to ischemia due to the compressive dressing. Emergent lateral escharotomies were performed, with immediate recovery of distal perfusion. One week afterward, the patient underwent surgical debridement of the burn on the dorsum of her finger and escharectomy of the ischemic eschar at the base. The lesions were covered with partial-thickness skin grafts. This case shows that acute compartment syndrome can lead to severe sequelae, such as the loss of an extremity or body segment. We must take utmost care in all our actions to avoid any (negligent) act that could lead to severe or permanent damage to our patients.

**New Insights and the Importance for the Pediatric Surgeon**

Iatrogenic compartment syndrome can lead to severe sequelae; it is infrequent, but preventable. Small children are at higher risk due to the difficulty of noncompliance. Pediatric surgeons must be aware of its etiology to prevent harm of their patients.

## Introduction

Acute compartment syndrome is a surgical emergency that can severely compromise the circulation, function, and even viability of an extremity. It occurs when an increase in the pressure within any closed, fixed compartment (defined by myofascial elements or bone) exceeds the perfusion pressure of the tissue, leading to ischemia and necrosis. 1 Most cases of acute extremity compartment syndrome develop after severe injuries such as fractures, but they can also occur after lesser injuries or, less frequently, they can be iatrogenic, secondary to tight dressings or tightly applied splints and casts, with the same terrible consequences. 2

We report a severe case of compartment syndrome due to a compressive burn dressing.

## Case Report

**Fig. 1:**
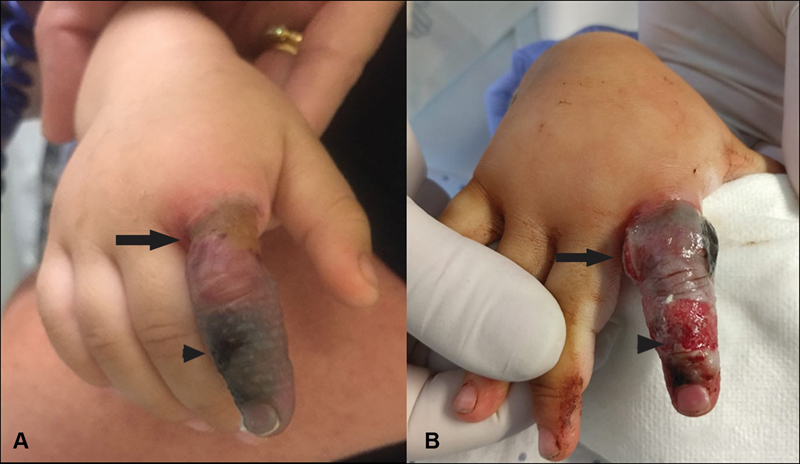
( **A** ) Ischemic circumferential constrictive eschar at the base of the finger (black arrow), secondary to compressive dressing, with severe vascular compromise of the finger. Arrowhead: burn lesion. ( **B** ) Immediate recovery of distal perfusion after emergent bilateral escharotomies (black arrow) were performed. Arrowhead: debrided burn.

**Fig. 2:**
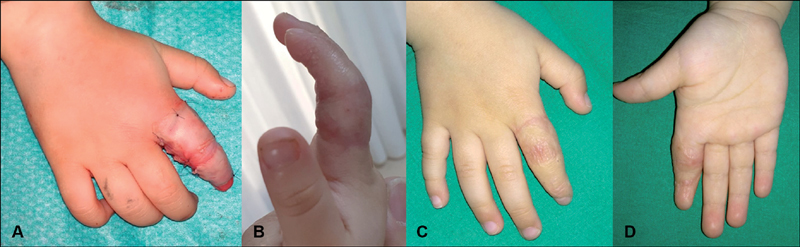
Treatment of the sequelae. ( **A** ) Immediate postoperative result after surgical debridement of the burn on the dorsum of the finger and escharectomy of the ischemic eschar at the base and grafting with partial-thickness skin grafts. ( **B** ) Severe palmar contracture of the digit at 3 months follow-up. ( **C** ) Dorsum of hand at 8 months follow-up, after surgically releasing the contracture and grafting with full-thickness skin graft. ( **D** ) Palm of hand.

An otherwise healthy 2-year-old girl arrived at her local health center with a contact burn with a hot iron several minutes before. She presented a noncircumferential, superficial partial-thickness burn on the dorsum of the mid phalanx of the second finger of her right hand. A compressive dressing was applied solely to the affected finger. Forty-eight hours afterward, the patient presented at the emergency room with severe pain of the finger. After removal of the dressing, a circumferential constrictive eschar was observed at the base of the finger, secondary to ischemia due to the compressive dressing, which generated a significant compartment syndrome with severe vascular compromise of the finger ( Fig. 1A ). Emergent bilateral escharotomies were performed, with immediate recovery of distal perfusion ( Fig. 1B ). One week afterward, the patient underwent surgical debridement of the burn on the dorsum of her finger and escharectomy of the ischemic eschar at the base. The lesions were covered with split-thickness skin grafts, the donor site being the ipsilateral arm ( Fig. 2A ). As soon as the graft healed, the patient was started on splinting and physical therapy. However, after 3 months, the patient presented with a palmar contracture of the digit that limited full extension of the finger ( Fig. 2B ). The contracture was surgically released and the resulting skin defect was covered with full-thickness skin graft (donor site: contralateral groin). At 8 months follow-up, and after intense splinting and physical therapy, the patient shows a normal function, achieving complete extension of the finger, with a mature, asymptomatic, flat scar ( Fig. 2C and D ).

## Discussion

Acute compartment syndrome is a devastating diagnosis that can lead to severe sequelae, such as the loss of an extremity or body segment. 3

Any pathologic condition that increases the volume inside a closed compartment (intrinsic causes, such as bleeding or edema after a fracture) or limits the external dilation of the compartment (extrinsic causes, such as burns or tight dressings) will increase the internal pressure of the compartment, potentially leading to acute compartment syndrome, with tissue necrosis. 4 Intrinsic causes, such as fractures or severe crush injuries, are by far the most frequent cause of acute compartment syndrome. Infrequently, iatrogenic causes like compressive dressings or tight splints and casts can also produce severe damage. 5 The case of acute compartment syndrome we present in this article is distinctly of iatrogenic origin, consequence of a negligent act. The application of an excessively tight circumferential dressing around the base of the finger produced a local necrosis of the skin, which behaved like a circumferential third-degree burn, restraining the dilation of the finger and thus hindering appropriate perfusion.

All cases of iatrogenic compartment syndrome are potentially preventable and easy to avoid if health professionals are adequately trained and provide care to their best of their knowledge, being extremely cautious in all their actions. 6 With regard to the presented case, all damage could have been avoided if a noncompressive, noncircumferential dressing had been applied and the tip of the finger had been left exposed to monitor for possible vascular compromise.

Although this case is the consequence of a neglectful act, it is nonetheless interesting to point out that the patient presented other risk factors that might have favored the development of the compartment syndrome. First, the patient sustained a thermal burn. This lesion was certainly not the cause of the compartment syndrome, for it was neither circumferential nor deep, but it must have triggered a local inflammatory response that might have contributed to the onset or aggravation of the compartment syndrome. 7 Second, small children are especially vulnerable to iatrogenic complications, for they are frequently unable to cooperate due to their immature cognitive abilities and their limited verbal capacity sometimes precludes adequate communication. 8 For a start, it can be very challenging to apply an adequate dressing on a toddler, for the patient will not collaborate. Also, some alarm symptoms (such as intense pain) might be overlooked in young children due to their inability to provide clinical information, resulting in delays in diagnosis and treatment. In any case, care providers that frequently take care of burns and/or children should be appropriately qualified and be aware of the particular idiosyncrasy of minors.

Cases such as the one we present here should result in education and training of health personnel at all levels, with a very especial emphasis on prevention of iatrogenic complications; practitioners and nursing staff should be aware of the very severe consequences their neglectful actions may have on patients. As health-care providers, we have a responsibility to provide the best possible medical care.

## Conclusion

Iatrogenic compartment syndrome is an infrequent but preventable cause of possible severe damages, such as the loss of an extremity or body segment. It is fundamental that health professionals are aware of its etiologies and risk factors and take utmost care in all their actions, to avoid negligent acts that could lead to severe or permanent damage to patients.
